# Supplementary material for: Transcription factor DUO1 generated by neo-functionalization is associated with evolution of sperm differentiation in plants
Source: Nat Commun. 2018 Dec 11;9:5283. doi: 10.1038/s41467-018-07728-3 (PMC6290024; doi:10.1038/s41467-018-07728-3)
Supplement: Supplementary file 1 — Supplementary Information [file 41467_2018_7728_MOESM1_ESM.pdf]

Supplementary Information for **Transcription factor DUO1 generated by neo-functionalization is associated with evolution of sperm differentiation in plants**

Higo and Kawashima *et al.*

Supplementary Figures 1 to 7

Supplementary Tables 1 to 4

Supplementary References 1 to 7

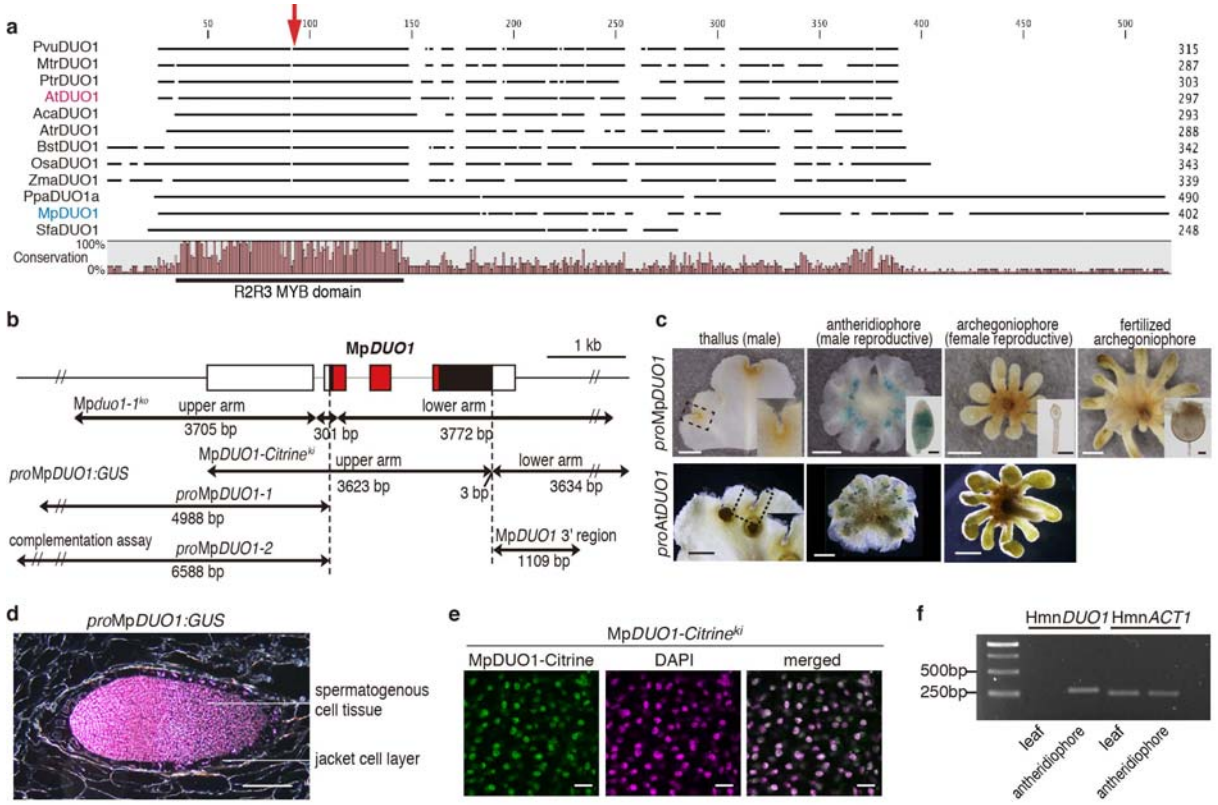

Supplementary Figure 1. Mp*DUO1* and Hmn*DUO1* Characterization. a, Schematic diagram of amino acid sequence alignment of the entire DUO1 protein from land plants: *Phaseolus vulgaris* (*Pvu*), *Medicago truncatula* (*Mtr*), *Populus trichocarpa* (*Ptr*), *Arabidopsis thaliana* (*At*), *Aquilegia caerulea* (*Aca*), *Amborella trichopoda* (*Atr*), *Brachypodium stacei* (*Bst*), *Oryza sativa* (*Osa*), *Zea mays* (*Zma*), *Physcomitrella patens* (*Ppa*), *Marchantia polymorpha* (*Mp*), and *Sphagnum fallax* (*Sfa*). The bottom vertical bars (pink) show the degree of conservation at each amino acid residue position. The red arrow indicates the position of DUO1-specific supernumerary lysine residue. The length of each DUO1 protein (number of aa) is indicated on the right. **b,** Schematic diagram of the Mp*DUO1* gene and the genomic fragments used in this study. White boxes represent UTRs, while black and red boxes represent coding exons. The sequence encoding the R2R3 MYB domain is shown in red. A 301-bp fragment between the upper and lower arms was replaced with a selection marker gene fragment in Mp*duo1-1*^{ko} plants.

c, GUS-stained *pro*Mp*DUO1:GUS* (top) and *pro*At*DUO1:GUS* (bottom) plants. From left to right, insets show an enlargement of the meristematic apical notch (dashed box), a dissected antheridium (where sperm cells are formed), an archegonium (where the egg is formed), and a sporophyte (the diploid body formed after fertilization). Antheridiophores and archegoniophores are male and female reproductive branches, respectively. **d,** Dark-field image of a section of an antheridium from a GUS-stained *pro*Mp*DUO1:GUS* plant. GUS staining is shown in pink. The jacket cells are a single layer of somatic cells that encloses the spermatogenous tissue which differentiate into sperm cells (see Fig. 1c). **e,** The subcellular localization of MpDUO1-Citrine fusion protein in developing sperm cells of the Mp*DUO1-Citrine*^{ki} plant. Citrine signal (left), DAPI-staining signal (middle), and merged (right) images are shown. **f,** Expression profiles of Hmn*DUO1,* the *DUO1* ortholog from *Haplomitrium mnioides,* which represents the most basal liverworts. Shoot tip region containing antheridia ("antheridiophore") and vegetative region devoid of antheridia ("leaf") were sampled from male plants. An actin gene (Hmn*ACT1*) was used as the internal control. Scale bars, 2 mm (c), 100 µm (c, insets), 100 µm. (d), and 5 µm (e).

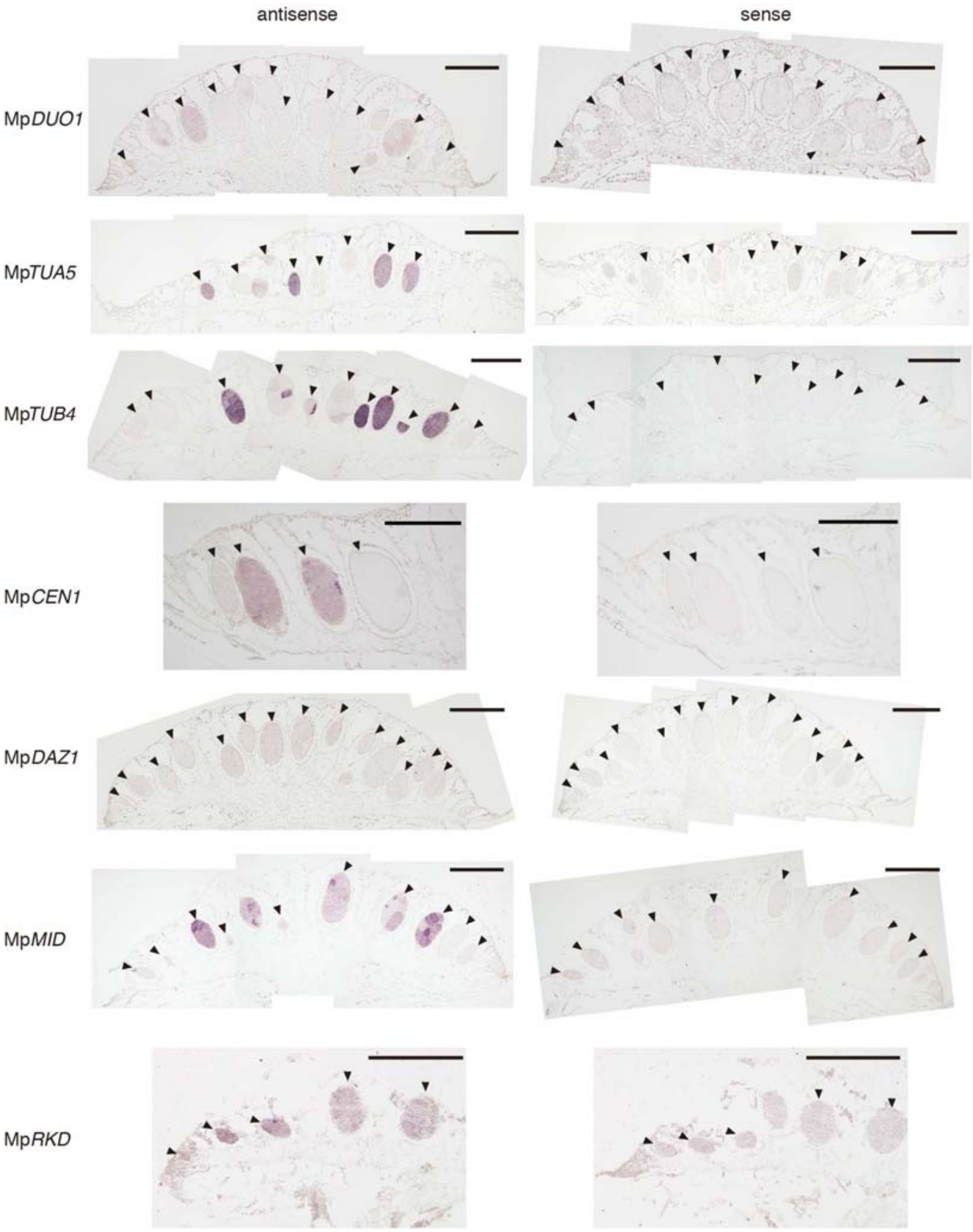

Supplementary Figure 2. Expression patterns of Mp*DUO1* and genes involved in sperm morphogenesis, the ortholog of At*DAZ1* and At*DAZ2*, and RWP-RK transcription factors in *Marchantia*. RNA *in situ* hybridization in developing antheridiophores of WT plants. hybridization images with antisense probes (left panels) and sense probes (right panels) from longitudinal serial sections are shown. Ellipsoid structures designated by arrowheads are

corresponding antheridia in consecutive sections hybridized with antisense or sense probe. Scale bars, 500µm.

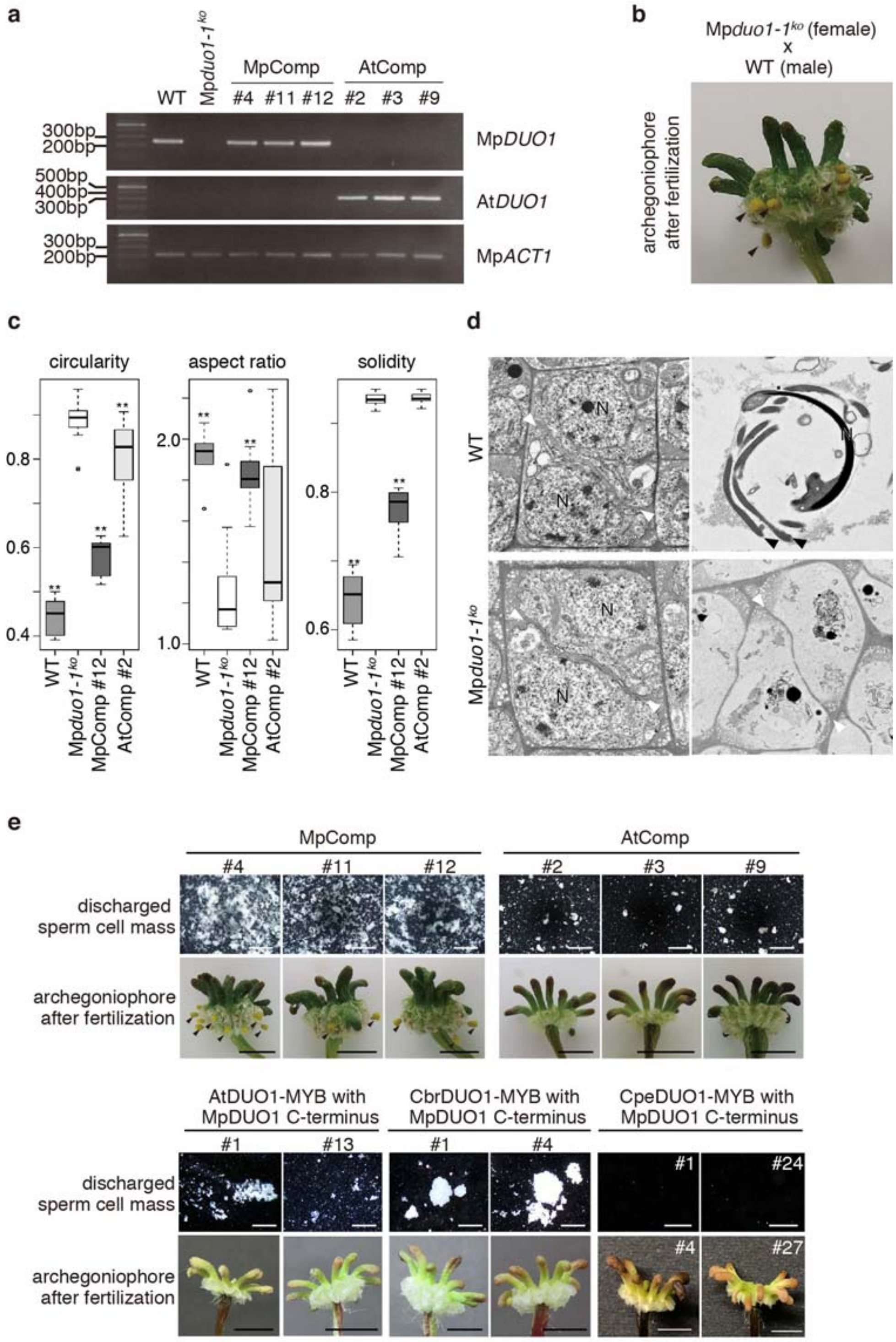

Supplementary Figure 3. Characterization of Mp*duo1-1*^{ko} plants. a, RT-PCR of Mp*DUO1* and At*DUO1* transcripts in antheridiophores of WT, Mp*duo1-1*^{ko}, and Mp*duo1-1*^{ko}

complemented by the Mp*DUO1* genomic fragment (MpComp) and by *pro*Mp*DUO1*:At*DUO1* (AtComp). Mp*ACT1* was used as the internal control. **b,** An archegoniophore from a female Mp*duo1-1*^{ko} line a month after crossing with a WT male. Successful fertilization results in mature yellow sporangia (arrows). **c,** Quantification of nuclear shape in mid-stage spermatids (see Fig. 2c; for description of developmental stages, see Fig. 1c). Nuclear shape characteristics (circularity, aspect ratio, and solidity) are shown as a box plot. Statistical significance relative to Mp*duo1-1*^{ko} was evaluated using Student's *t*-test (**, $p < 0.01$). Error bars indicate mean ± SD; $n > 15$ nuclei. **d,** Transmission electron micrographs of a pair of spermatids at early (left) and late (right) stages of spermiogenesis from WT (upper) and Mp*duo1-1*^{ko} (lower). White arrowheads indicate the cell wall between daughter cells generated by the final diagonal division. Black arrowheads indicate the flagella. Note that sperm cells abort in Mp*duo1-1*^{ko}. N, nucleus. **e,** Complementation of Mp*duo1-1*^{ko} with the Mp*DUO1* genomic fragment (MpComp), *pro*Mp*DUO1*:At*DUO1* (AtComp), *pro*Mp*DUO1*:*AtChimera* (AtDUO1-MYB with MpDUO1 C-terminus), *pro*Mp*DUO1*:*CbrChimera* (CbrDUO1-MYB with MpDUO1 C-terminus), and *pro*Mp*DUO1*:*CpeChimera* (CpeDUO1-MYB with MpDUO1 C-terminus). Schematic diagrams of the three chimeric constructs are shown in Fig. 7c, d. The top panels show sperm masses (white) discharged from mature antheridiophores into water. The bottom panels show the archegoniophore of WT (female) about a month after crossing with each line. Black arrows point to the mature yellow sporangia. Scale bars, 5 mm (b), 2 µm (d), 400 µm (e, top), and 5 mm (e, bottom).

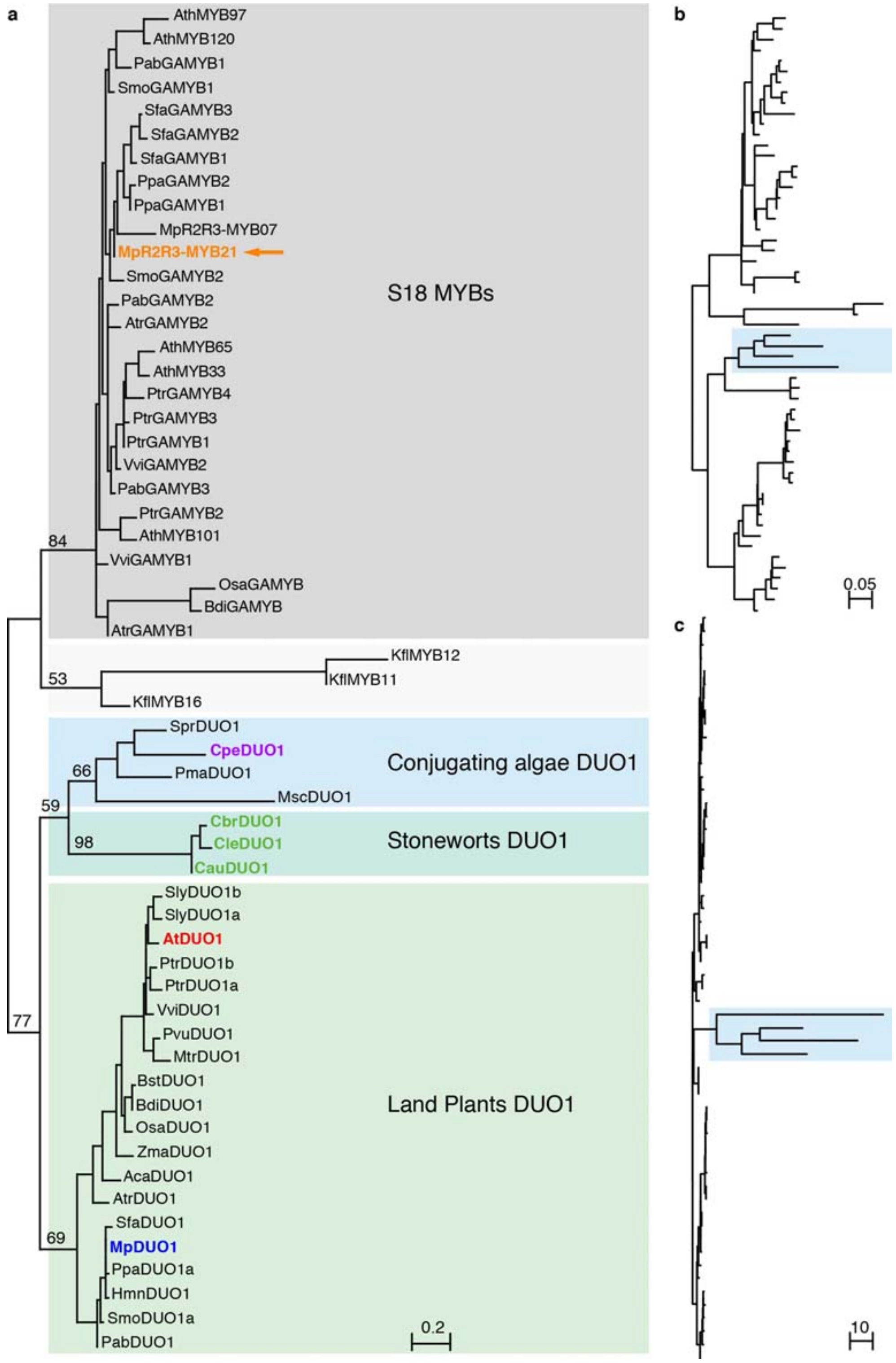

Supplementary Figure 4. Selection pressure on *DUO1* orthologs. a, Maximum likelihood tree of MYB domain equences from DUO1 orthologs and S18 MYBs. Highlighted boxes indicate

sequences of related DUO1 orthologs evolving under distinct selection pressures (ω=dN/dS), as determined by likelihood ratio tests of different models. Values of ω are comparable for DUO1 orthologs in stoneworts (0.0645) and land plants (0.0295). Maximum likelihood bootstrap support for relevant clades indicated on branches. Scale indicates substitutions per site. **b,** dN tree showing ratios of nonsynonymous mutations per nonsynonymous site used in ω (dN/dS)calculations. Scale indicates dN values per branch. The highlighted box indicates the conjugating green algal DUO1 clade. **c,** dS tree showing ratios of synonymous mutations per synonymous site used in ω (dN/dS) calculations. Scale indicates dS values per branch. The highlighted box indicates the conjugating green algal DUO1 clade. The low ω (0.0029) in conjugating green algae is caused by a high synonymous substitution (dS) rather than a decrease in non-synonymous substitution (dN) (compare b and c). This might be explained by a high mutation rate in DUO1 orthologs in conjugating green algae[1, 2] or by changes in selective pressure related to decreased protein levels[3]. See details in Supplementary Table 2.

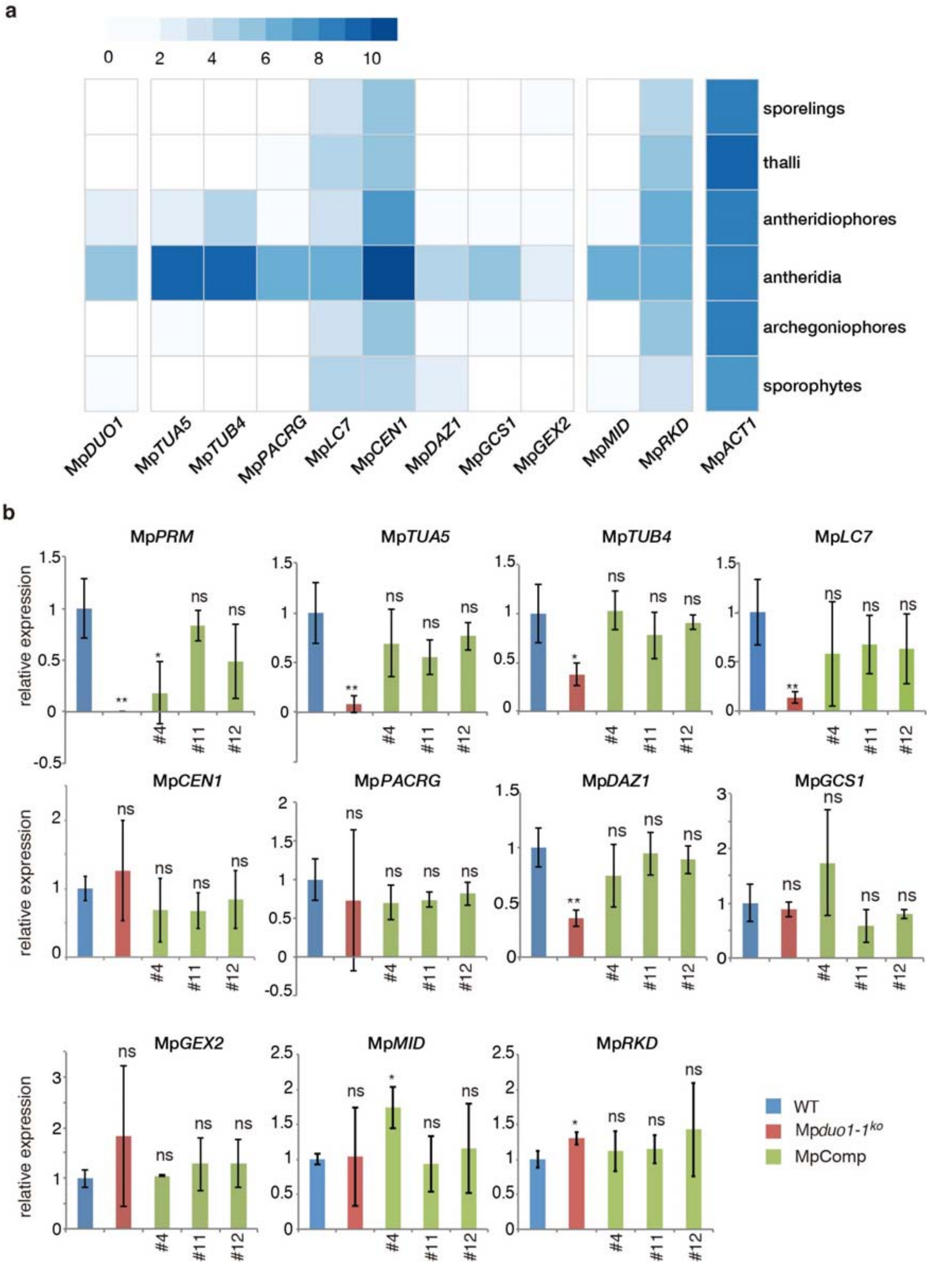

Supplementary Figure 5. Characterization of putative DUO1 target genes in *Marchantia.* a,

Expression profiles of genes involved in *Marchantia* sperm morphogenesis, the orthologues of

AtDUO1 target genes, and an actin gene (Mp*ACT1*) based on RNA-seq data from major tissue types that mark key steps during the *Marchantia* life cycle (see ref. [4]). Sporelings are formed after germination of spores and develop to somatic thalli. Upon induction of sexual reproduction, thalli produce reproductive branches (antheridiophores in male and archegoniophores in female). Antheridia host differentiating sperm cells. Sperm fertilize an egg to produce the diploid sporophyte developing on archegoniophores. Meiosis takes place inside the part of sporophyte (sporangia, see Fig. 2b) to produce haploid spores. Heatmap values are shown as $\log_2$-normalized TPM (Transcript Per Million). **b,** Quantitative real-time PCR analysis of transcripts of flagella component genes, a PROTAMIN-LIKE gene (Mp*PRM*), the orthologues of AtDUO1 target genes, and RWP-RK transcription factor genes in stage 4 antheridiophores. The relative expression of each gene in Mp*duo1-1*^{ko} and MpComp lines was compared with WT. Mp*ACT1* was used as an internal control. Statistical significance relative to WT was evaluated using Student's *t*-test (**, $p < 0.01$; *, $p < 0.05$). Error bars indicate mean ± SD; n = 3.

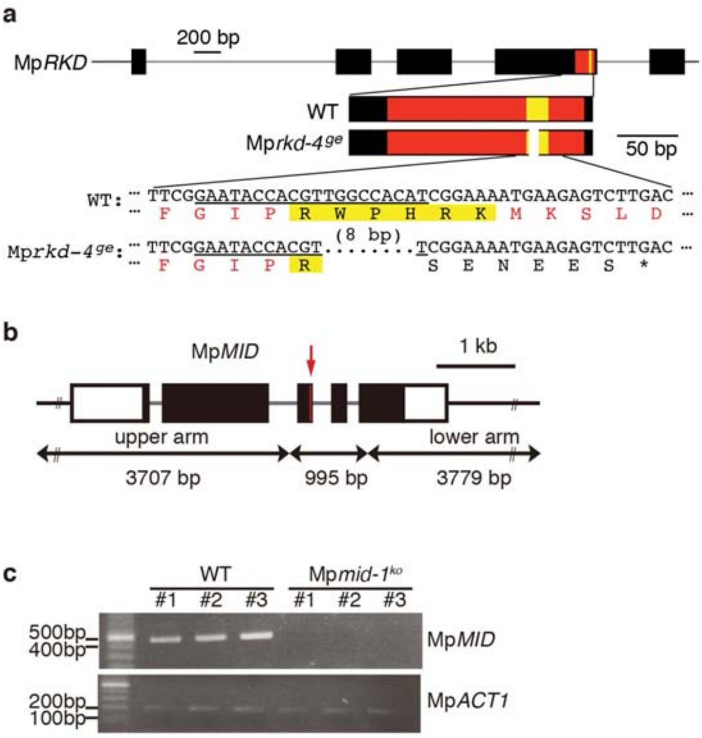

Supplementary Figure 6. Characterization of two RWP-RK transcription factor genes, Mp*MID* and Mp*RKD*, in *Marchantia*. a, Schematic diagram of the Mp*RKD* gene and mutation in Mp*rkd-4*^{ge}. Black boxes represent coding exons and grey bars represent introns. The red box represents the conserved RWP-RK DNA binding domain while the yellow box indicates the RWP-RK motif. A part of nucleotide and corresponding amino acid sequences are depicted below showing an sgRNA target sequence (underlined) and mutational changes in Mp*rkd-4*^{ge}. **b,** Schematic diagram of the Mp*MID* gene and the genomic fragments used in this study. White and black boxes represent the 5′ and 3′ UTR and coding exons, respectively, while gray bars represent introns. The RWP-RK DNA binding motif is shown in red (red arrow). A 995-bp fragment between the upper and lower arms was replaced with a selection marker gene fragment in Mp*mid-1*^{ko} plants. **c,** RT-PCR detection of Mp*MID* transcripts at stage 4 antheridiophores of WT and Mp*mid-1*^{ko}. Mp*ACT1* was used as an internal control.

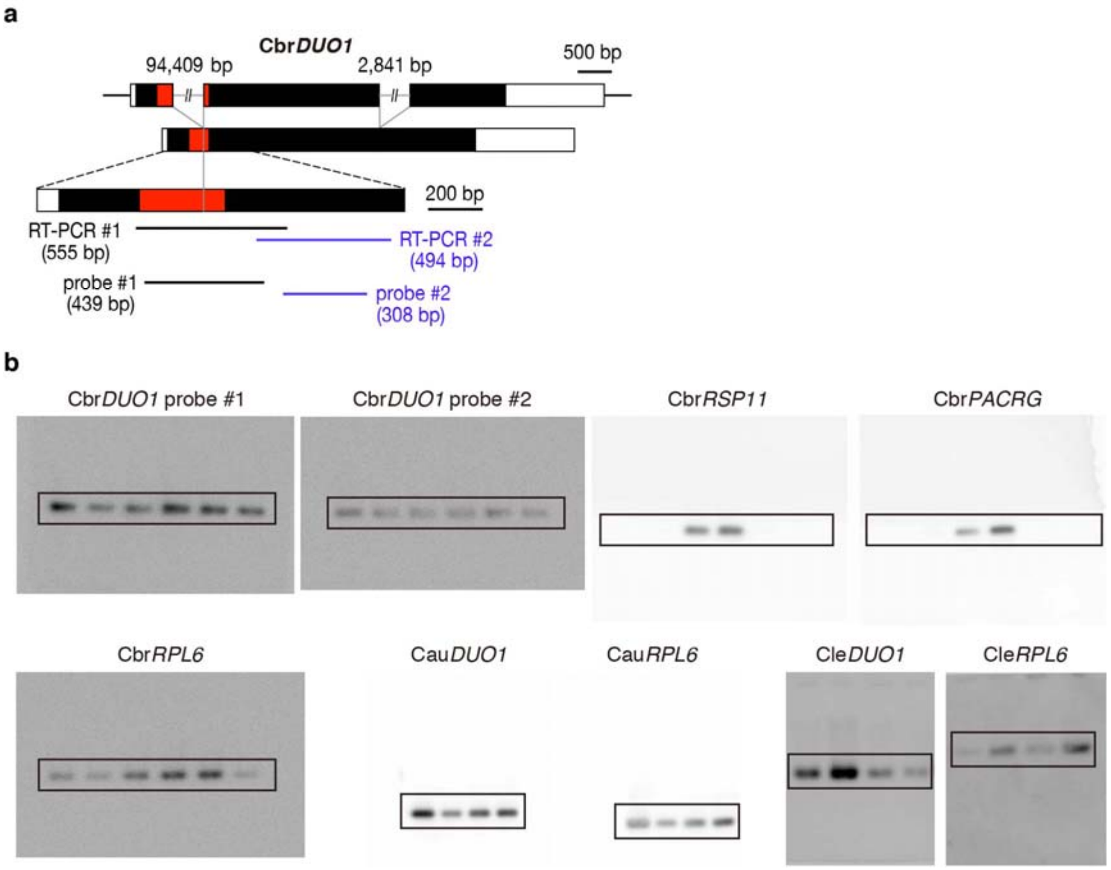

Supplementary Figure 7. Details of RT-PCR Southern blotting analysis of *Chara* genes. a, Schematic diagram of gene structure (upper) and transcript (middle) of the *DUO1* ortholog from *Chara braunii* (Cbr*DUO1*). The color codes used are as in Supplementary Fig. 1b. Two RT-PCR products and probes used for Southern hybridization in Fig. 7a are shown. **b,** Whole untrimmed images of Southern blotting shown in Fig. 7a.

Supplementary Table 1. Promoter analysis of DUO1 target genes in land plants.

	Species	*DAZ1* promoter		*GCS1/HAP2* promoter		*GEX2* promoter	
		gene name	number of DBS[a]	gene name	number of DBS[a]	gene name	number of DBS[a]
Angiosperms	*Arabidopsis thaliana*	At*DAZ1*<br>At*DAZ2*	2<br>1	At*GCS1/HAP2*	3	At*GEX2*	2
	Salix purpurea	SapurV1A.0125s0370<br>SapurV1A.0374s0220<br>SapurV1A.0475s0070<br>SapurV1A.0530s0140<br>SapurV1A.3538s0020	1<br>1<br>1<br>1<br>1	SapurV1A.0507s0190	1	SapurV1A.1573s0050	3
	Eucalyptus grandis	Eucgr.I01518	2	Eucgr.D01915	3	Eucgr.H03956	3
	Medicago truncatula	Medtr5g062300<br>Medtr5g099040	3<br>2	Medtr5g010740	3	Medtr2g436170<br>Medtr2g435850<br>Medtr5g020160	1<br>1<br>2
	Solanum lycopersicum	Solyc06g060480.1	1	Solyc08g080450.1	1	Solyc01g066510.1	2
	Aquilegia coerulea Goldsmith	Aquca_015_00379	1	Aquca_013_00398	3	Aquca_003_00691	1
	Zea mays	GRMZM2G132057	2	GRMZM2G412911	4	GRMZM2G036832	2
	Oryza sativa	Os02g19200	3	LOC_Os05g18730<br>LOC_Os09g35720	1<br>1	LOC_Os09g25650	3
	Amborella trichopoda	scaffold00001.9	2	scaffold00069.68<br>scaffold00069.69	0<br>1	scaffold00019.109	2
Lycophytes	*Selaginella moellendorffii*	410896<br>424361	3<br>4	XP_2971451.1	0	416480<br>407438<br>412212	0<br>1<br>1
Bryophytes	*Physcomitrella patens*	Pp3c22_2200<br>Pp3c19_21390<br>Pp3c21_440<br>Pp3c18_22010	1<br>0<br>0<br>0	Pp3c25_15230	0	---[b]	
	Sphagnum fallax	Sphfalx0048s0033<br>Sphfalx0022s0120<br>Sphfalx0020s0104<br>Sphfalx0179s0034<br>Sphfalx0019s0188	2<br>1<br>1<br>2<br>1	---[b]		---[b]	
	Marchantia polymorpha	Mp*DAZ1*	1	Mp*GCS1*	0	Mp*GEX2*	0

a: DBS means DUO1 binding motif (5´-RRCSGTT-3´)

b: no orthologous gene

Supplementary Table 2. Likelihood ratio tests and selection pressure analyses of S18 MYB and DUO1 transcription factors.

Branch Model model=2, NSsites=0, cleandata=0

	lnL	df	p-value	
Test 1 (3 ω ratios)	-8207.425128	2	0.0292	*
Test 2 (4 ω ratios)	-8207.139007	3	0.0541	
Test 3 (4 ω ratios)	-8204.454659	3	0.0046	*
Test 4 (4 ω ratios)	-8205.166649	3	0.0089	*
Test 5 (5 ω ratios)	-8201.704103	4	0.0009	*
Test 6: Test 3/Test 1	-8204.454659	1	0.0147	*
Test7: Test 4/Test 1	-8205.166649	1	0.2327	
Test8: Test 5/Test 3	-8201.704103	1	0.019	*

Test 1:

**model=2 NSsites=0**	Background	S18	DUO1
ω for branches	0.0816	0.0337	0.0304

lnL0 =-8210.956591

lnL1 =-8207.425128

Test 2:

**model=2 NSsites=0**	Background	S18	Algae DUO1	Land plant DUO1
ω for branches	0.0808	0.0338	0.0342	0.0297

lnL0 =-8210.956591

lnL1 =-8207.139007

Test 3:

**model=2 NSsites=0**	Background	S18	Zyg DUO1	Land + Chara DUO1
ω for branches	0.0821	0.0338	0.0029	0.0321

lnL0 =-8210.956591

lnL1 =-8204.454659

Test 4:

**model=2 NSsites=0**	Background	S18	Chara DUO1	Land + Zyg DUO1
ω for branches	0.0821	0.0337	0.0617	0.0282

lnL0 =-8210.956591

lnL1 =-8205.166649

Test 5:

**model=2 NSsites=0**	Background	S18	Zyg DUO1	Chara DUO1	Land DUO1
ω for branches	0.0856	0.0338	0.0029	0.0645	0.0295

lnL0 =-8210.956591

lnL1 =-8201.704103

Test9: Branch-sites Model model=2, NSsites=2, cleandata=0

	**# parameters**	**lnL**	**df**	**p-value**
Control6 (ZygDUO1) fix w2=1		-8199.282529		
Test6 (ZygDUO1)	4	-8198.29907	1	0.1607

dN/dS(w) for site classes (K=4)				
site class	0	1	2a	2b
proportion	0.81221	0	0.18779	0
background w	0.03335	1	0.03335	1
foreground w	0.03335	1	3.7376	3.7376

lnL0 = -8199.282529

lnL1 = -8198.299070

Notes: **Test1**, Test of two clades hypothesis (S18 MYBs and DUO1) compared to null hypothesis M0. Alternative hypothesis found to be more likely ($p<0.05$); **Test2**, Test of three clades hypothesis (S18 MYBs, Stonewort + conjugating green algal DUO1, Land plant DUO1)

compared to null hypothesis. Alternative hypothesis not found to be more likely ($p>0.05$); **Test3**, Test of three clades hypothesis (S18 MYBs, Conjugating green algal DUO1, Stonewort + Land plant DUO1) compared to null hypothesis. Alternative hypothesis found to be more likely ($p<0.05$); **Test4**, Test of three clades hypothesis (S18 MYBs, Land plant + Conjugating green algal DUO1, Stonewort DUO1) compared to null hypothesis. Alternative hypothesis found to be more likely ($p<0.05$); **Test5**, Test of four clades hypothesis (S18 MYBs, Conjugating algal DUO1, Stonewort DUO1, Land plant DUO1) compared to null hypothesis. Alternative hypothesis found to be more likely ($p<0.05$); **Test6**, Test of three clades hypothesis (S18 MYBs, Conjugating green algal DUO1, Stonewort + Land plant DUO1) compared to two clades hypothesis (S18 MYBs and DUO1). Alternative hypothesis found to be more likely ($p<0.05$); **Test7**, Test of three clades hypothesis (S18 MYBs, Conjugating green algal + Land plant DUO1, Stonewort DUO1) compared to two clades hypothesis (S18 MYBs and DUO1). Alternative hypothesis found to be more likely ($p<0.05$); **Test8**, Test of four clades hypothesis (S18 MYBs, Conjugating green algal DUO1, Stonewort DUO1, Land plant DUO1) compared to three clades hypothesis (S18 MYBs, Conjugating green algal DUO1, Stonewort + Land plant DUO1). Alternative hypothesis found to be more likely ($p<0.05$); **Test9**, Test for positive selection acting on sites in the Conjugating green algal DUO1 clade. No significant sites were found ($p>0.05$).

Supplementary Table 3. Oligonucleotide and primer sequences used for vector construction and PCR.

fragment name	primer sequence	
**vector constraction**		
Mp*duo1-1*^{ko} upper arm	ttatccctaggcgcgGTTTAGTAAGAAATAATGACATAATGACTTGC	aacactagtggcgcgTCTTCGAACTAAGAGGTTGTCAGATGAAAG
Mp*duo1-1*^{ko} lower arm	ccgggcaagctttaatCCCTTCCGGAATACGTAGCGAGTA	ctaaggtagcgattaatCTTCACCTTTCGAGGTGGAGTTGC
Mp*DUO1-Citrine*^{ki} upper arm	ctaaggtagcgattaCTCTCCTCTGCTGCCTTTGG	gcctccaagcttaatACTCAGTCGGGCAACCCAC
Mp*DUO1-Citrine*^{ki} lower arm	taaactagtggcgcgTAGGACGAGGATACTAGCGAAAAGG	ttatccctaggcgcgGGATCGTGGTTGGCACTCATATTC
Mp*mid-1*^{ko} upper arm	ttatccctaggcgcgAGTCGTCCGGAGAGGAACTAAAACCAG	taaactagtggcgcgCTTGTGGAGCTAGAGCGCGCTATG
Mp*mid-1*^{ko} lower arm	gcccgggcaagcttaGACCTCAATATGGACAGTAGAGATAGTG	ctaaggtagcgattaTCTGAATGTGTAATTTCGGGGAACAATGTC
proMp*DUO1-1*	atccggtaccgaattGGAAAATTTTGTAGATGATTATAATTGGTATG	tgcggccgcgaattcTTTTTATCTGAGACCAGCCACTCC
proMp*DUO1-2*	aaccaattcagtcgacCTGACTAGTGTGGATTGTGATGC	attcggtaccggatccCATTTTTTATCTGAGACCAGCCACTC
3' reiogn *MpDUO1*	TAGGACGAGGATACTAGCGAAAAGG	TTGTCACTCTGATCTCTCTAATTGG
gMp*DUO1-HA-FLAG*-1	ggggacaagtttgtacaaaaaagcaggctccCTGACTAGTGTGGATTGTGATGC	aagctgtcccACTCAGTCGGGCAACCCA
gMp*DUO1-HA-FLAG*-2	TGGGTTGCCCGACTGAGTgggacagctttcttgtac	GGGGACCACTTTGTACAAGAAAGCTGGGTCTTAggggacaactttgtataata
At*DUO1* ORF	ctcagataaaaaatgGAAGCGAAGAAGGAAGAGATAAAG	atctcgagtgcggccgcCTAAGGACTTGGGATTGGATCAAC
Mp*DUO1* ORF	ggggacaagtttgtacaaaaaagcaggctccATGAAAACGATTCAAAACGGA	ggggaccactttgtacaagaaagctgggtcTTAACTCAGTCGGGCAAC
At*DUO1*-Cter	GCACTGCATAACTCCTCTGATGCA	ggggaccactttgtacaagaaagctgggtccTAAGGACTTGGGATTGGATCA
At*DUO1*-MYB for Chimera DUO1	ctcagataaaaaATGGAAGCGAAGAAGGAAGAGATAAAG	ggcattttaggacgctgcagAATCCTAGCGAGTCTCTTTTG
Mp*DUO1* C-terminus for Chimera DUO1	CTGCAGCGTCCTAAAATGCC	atctcgagtgcggccgcTTAACTCAGTCGGGCAACCC
Cbr*DUO1*-MYB for Chimera DUO1	ctcagataaaaaATGGACTCCGGCGGAGGAGC	CAGCTTCTTCATCCGCATATTCCAG
Cpe*DUO1*-MYB for Chimera DUO1	ctcagataaaaaATGGAGAACGGCAGCAGCAGC	tgcacctcaagaggcaggccCTGCAGCGTCCTAAAATGCC
proAt*DUO1*	ggggacaactttgtatagaaaagttgCTGGAAGTTTGTTGTTGAGAG	ggggactgctttttgtacaaacttggTTTCCTCATCGCTAATCGATC
mRuby2	ggggacagctttcttgtacaaagtgggtATGGTGTCTAaGGGCGAAGAG	ggggacaactttgtataataaagttggTTACTTGTACAGCTCGTCCATCC
proAt*DUO1* for proAt*DUO1:GUS*	caccCTGGAAGTTTGTTGTTGAGAGAGC	TTTCCTCATCGCTAATCGATCTCTC
Chimera 1 for trans-activation	ggggacaagtttgtacaaaaaagcaggctccATGAAAACGATTCAAAACGGA	ggggaccactttgtacaagaaagctgggtccTAAGGACTTGGGATTGGATCA
Chimera 2 for trans-activation	ggggacaagtttgtacaaaaaagcaggctccATGGAGACCATGGAACTAGGCAG	ggggaccactttgtacaagaaagctgggtccTAAGGACTTGGGATTGGATCA
Chimera 3 for trans-activation	ggggacaagtttgtacaaaaaagcaggctccATGAAAACGATTCAAAACGGA	ggggaccactttgtacaagaaagctgggtccTAAGGACTTGGGATTGGATCA
Chimera 4 for trans-activation	ggggacaagtttgtacaaaaaagcaggctccATGAAAACGATTCAAAACGGA	ggggaccactttgtacaagaaagctgggtccTAAGGACTTGGGATTGGATCA
Chimera 5 for trans-activation	ggggacaagtttgtacaaaaaagcaggctccATGAAAACGATTCAAAACGGA	ggggaccactttgtacaagaaagctgggtccTAAGGACTTGGGATTGGATCA
Chimera 6 for trans-activation	ggggacaagtttgtacaaaaaagcaggctccATGAAAACGATTCAAAACGGA	ggggaccactttgtacaagaaagctgggtccTAAGGACTTGGGATTGGATCA
Chimera 7 for trans-activation	ggggacaagtttgtacaaaaaagcaggctccATGAAAACGATTCAAAACGGA	ggggaccactttgtacaagaaagctgggtccTAAGGACTTGGGATTGGATCA
guideRNA for Mp*rkd-4*^{ge}	ctcgGAATACCACGTTGGCCACAT	aaacATGTGGCCAACGTGGTATTC
**genetic screening**		
Mp*duo1-1*^{ko}-1	cggatccGGAAAATTTTGTAGATGATTATAATTGGTATG	TGCCAGAAGGACTATGCACCTG
Mp*duo1-1*^{ko}-2	GAGACGGTCCATGGATGACTAG	TGCCAGAAGGACTATGCACCTG
Mp*mid-1*^{ko}-1	CCTTGTTCCAAAGAGTTCCGTGAG	CCATTAGGTAATGGTCTCAGCCTTG

Mp*mid-1*^{ko}-2	CACCTGCCCTACAAGAAACCATGA	ctaaggtagcgattaTCTGAATGTGTAATTTCGGGGAACAATGTC
Mp*DUO1-Citrine*^{ki}	GACCCCAGCATTCAAGAACCTG	CAGGTGGAAGAATCTAAGAATCAACC
Mp*rkd-4*^{ge}	TCCTCAGGCGACAAAGTTGAGG	AGCATTGTGAATTCTCCATTAGTCGTGA
**probes for RNA in situ hybridization**		
Mp*DUO1*	CTCTTAGTTCGAAGAACAGCGGAG	GACGGCACTAATCCTGTGCCAG
Mp*TUA5*	GAGAATTCTCCGAAGCTCGTGAG	GTCAGCTGAGATTGCACTCATGCTC
Mp*TUB4*	GAGAGCAACATGAACGATCTTGTG	CTCCGTGTTTGGTAATCCTCCTC
Mp*CEN1*	CGAGGCTTTTGACCTCTTTG	ccgctcgagtAAAGAGGCTTGTCTTTTTCATGA
Mp*DAZ1*	CAACCGAGGGATTATCAACTCATATTGC	GCCTTCGTTTGATGACCAGGAG
Mp*MID*	CAGATTTGTACAAAAGGCTGTACTCCAG	CAGCCATTAGGTAATGGTCTCAGC
Mp*RKD*	ATGGGAAGCACCGGCCTGCG	AGTGATCTCGGTCATCCGCTC
**probes for southern hybridization**		
Cbr*DUO1* probe#1	GTATGGCTGCCTGAGGAAGACG	CTACATCTCCAGGTGATGCCAGCATG
Cbr*DUO1* probe#2	CTGTTCCTGCTCATCATCACC	GTGGTGGTGAGGAGAGAGATG
Cbr*RSP11*	TCCTGGAGTTTTCCGCTCAC	TCATCTTTTGCGCCTCGTCT
Cbr*PACRG*	CTTCGACCAGACGCTACCTG	AGCTGAGGAATAACGGGCAG
Cbr*RLP6*	CAAGTTCCCTGTTCATGCCAAG	GTCGACGTCGCAATCACGTAG
Cau*DUO1*	GTATGGCTGCCTGAGGAAGATG	CTACATCTCCAGGTGATGCCAGCATG
Cau*RPL6*	GTTCCCTGTTCACGCCAAGAAG	GTCGACGTCGCAATCACGTAG
Cle*DUO1*	GATTTTGAAGGGATACGTGGCCAATC	CTCCAACCTTGCTAGCTTTGCTAG
Cle*RPL6*	ACGATGTGCCCAAGCCACGTC	TTTCTCGATCACTGGAAGTAGC
**RT-PCR**		
Mp*ACT1*	CAGTGTCTGGATCGGAGGAT	CACCACTCAAACTGGAAGCA
Mp*PRM*	ATCCAGCGGCGTGAGCCAGA	CGAAGAATGCGGAAGACTGA
Mp*TUA5*	GTGCAGATCGACGACAAAGGATC	GAGAATTCTCCGAAGCTCGTGAG
Mp*TUB4*	CACCGTTGCTCGAGCCAATC	GAGAGCAACATGAACGATCTTGTG
Mp*LC7*	CGTCGACAATGACGGAAATA	TACTGTTGTGCGACCAGGAG
Mp*CEN1*	CCAAAATGGGTGAACGAGAT	ACCATCTCGATCTGCTTCGT
Mp*PACRG*	CTTGCCAGTGTTTTTCGATG	CGGAGGGATCTCTTGTGTTC
Mp*DAZ1*	TGTTTTATCACCCGCCTAGC	AGGCGAACAAAACATTGGAC
Mp*GCS1*	ACGAAGTGGTTCGGCGTATT	GTGGTCACGTAGCACTTGAAGTC
Mp*GEX2*	AGCAGATGGTAACCCACAGG	AGGATCAGGACCACCAGATG
Mp*MID*	CACATCATCTGAAGCTGTCAGATCTATC	CTAAACTTCGAACCTTGCGTCCTGG
Mp*RKD*	TCGAGCTTTGGCAATGCATA	TTGCGGATTCCTCGTGACAC
Mp*DUO1*	CTCTTAGTTCGAAGAACAGCGGAG	CACCCACTCTTGAGATCCGGCTTG
At*DUO1*	AACGTCAAACCAATCCGTCAATCC	CGAACAATGGCTCAGAAGAATCAGC
Hmn*ACT1*	TTCTCGCACCAGCTCCTCGC	TACGATCCGCAATACCAGGG
Hmn*DUO1*	GCCGGGTAGGAGTAATTGTTAAGC	CCACCACTAGTTTTTCCTCCTCTG
Cbr*DUO1* probe#1	GTCCAAAGCGCCCCTTGAAG	GGTGATGATGAGCAGGAACAG
Cbr*DUO1* probe#2	CATGCTGGCATCACCTGGAGATGTAG	CAGCACTGGTAGGTGAGACAC
Cbr*RSP11*	ACTGCGTGGAGCAGATTAGA	GGCGTTCAAGAAAGTTGGGT
Cbr*PACRG*	ATGCTTCGACCAGACGCTAC	CCGTGCACGTGTTTTTCTGT
Cbr*RLP6*	CTCCGTCATGTACGGCAAGAGAG	CCTTCTGATCGTCCTTCCTCTC
Cau*DUO1*	CTGTTCCTGCTCATCATCACC	GATGGATATGGTGGTGATGAGGAG
Cau*RPL6*	CTCCGTCATGTACGGCAAGAGAG	CCTTCTGATCGTCCTTCCTCTC
Cle*DUO1*	GTCCAAAGCGCCCCTTGAAG	CTACATCTCCAGGTGATGCCAG
Cle*RPL6*	AAGTTGCCTAAGTTCTACCCCG	AAGTTCATGGGGCTTCATGCCG
**fragments synthesized by a company**		
Chimera 1 Nter for trans-activation	ATGAAAACGATTCAAAACGGAGATGAAGTTCTACGGAAGGGTCCATGGATGCCCGAAGAAGACGAAATCCTGGTGGAGTATGTGCGACAATTT GGTGCACGAGACTGGAGTTCCATTCGTTCCAAAGGCTTGTTGCCTCGTACGGGAAAATCTTGCCGTCTGCGATGGGTCAACAAACTCAAGCC GGATCTCAAGAGAAGTGGGTGTAAATTTTCCCCCGAGGAAGAGAAATTGGTAGTGGAGATGCAGGCGAAGCTTGGTAACAAGTGGGCAAAAA TTGCTTCATGTCTTCCTGGACGTACCGACAACGATGTGAAAAATTTCTGGAGCACCCGGCAGAAACGCATATTGCGCGCACTGCATAACTCCT CTGATGCA	
Chimera 2 Nter for trans-activation	ATGGAGACCATGGAACTAGGCAGCGCTCCGGATTTCTCTGAGGATGTTGGAGCACTAAAGAAGGGACCGTGGACATCGGCCGAAGACGCGA TTCTCGTAGCTTATGTCACCAAGCACGGCGAAGGAAACTGGAACTCGGTGCAGAAGCATTCGGGCCTGTACCGCTGCGGCAAAAGCTGCCG CCTGCGCTGGGCCAATCACCTCCGTCCCAATCTCAAGAAGGGAGCCTTCACACCGGAGGAGGAGCGGATGATTATCGAACTGCACGCCAAG CTTGGCAACAAATGGGCTCGTATGGCTGCCCAGTTGCCAGGTCGCACGGACAACGAAATTAAGAACTACTGGAACACTCGGATCAAGCGGAG GATGCGAGCACTGCATAACTCCTCTGATGCA	

Chimera 3 Nter for trans-activation	ATGAAAACGATTCAAAACGGAGATGAAGTTCTACGGAAGGGTCCATGGATGCCCGAAGAAGACGAAATCCTGGTGGAGTATGTGCGACAATTT<br>GGTGAAGGAAACTGGAACTCGGTGCAGAAGCATTCGGGCTTGCCTCGTACGGGAAAATCTTGCCGTCTGCGATGGGTCAACAAACTCAAGC<br>CGGATCTCAAGAGAAGTGGGTGTAAATTTTCCCCCGAGGAAGAGAAATTGGTAGTGGAGATGCAGGCGAAGCTTGGTAACAAGTGGGCAAAA<br>ATTGCTTCATGTCTTCCTGGACGTACCGACAACGATGTGAAAAATTTCTGGAGCACCCGGCAGAAACGCATATTGCGCGCACTGCATAACTCC<br>TCTGATGCA
Chimera 4 Nter for trans-activation	ATGAAAACGATTCAAAACGGAGATGAAGTTCTACGGAAGGGTCCATGGATGCCCGAAGAAGACGAAATCCTGGTGGAGTATGTGCGACAATTT<br>GGTGCACGAGACTGGAGTTCCATTCGTTCCAAAGGCTTGTTGCCTCGTACGGGAAAATCTTGCCGTCTGCGATGGGTCAACAAACTCAAGCC<br>GGATCTCAAGAGAAGTGCCTTTTCCCCCGAGGAAGAGAAATTGGTAGTGGAGATGCAGGCGAAGCTTGGTAACAAGTGGGCAAAAATTGCTT<br>CATGTCTTCCTGGACGTACCGACAACGATGTGAAAAATTTCTGGAGCACCCGGCAGAAACGCATATTGCGCGCACTGCATAACTCCTCTGATG<br>CA
Chimera 5 Nter for trans-activation	ATGAAAACGATTCAAAACGGAGATGAAGTTCTACGGAAGGGTCCATGGATGCCCGAAGAAGACGAAATCCTGGTGGAGTATGTGCGACAATTT<br>GGTGCACGAGACTGGAGTTCCATTCGTTCCAAAGGCTTGTTGCCTCGTACGGGAAAATCTTGCCGTCTGCGATGGGTCAACAAACTCAAGCC<br>GGATCTCAAGAGAAGTGGGTGTAAATTTTCCCCCGAGGAAGAGAAATTGGTAGTGGAGATGCAGGCGAAGCTTGGTAACAAGTGGGCAAAAA<br>TTGCTTCATGTCTTCCTGGACGTACCGACAACGAAATTAAGAACTACTGGAACACTCGGATCAAGCGGAGGTTGCGCGCACTGCATAACTCCT<br>CTGATGCA
Chimera 6 Nter for trans-activation	ATGAAAACGATTCAAAACGGAGATGAAGTTCTACGGAAGGGTCCATGGATGCCCGAAGAAGACGAAATCCTGGTGGAGTATGTGCGACAATTT<br>GGTGCACGAGACTGGAGTTCCATTCGTTCCAAAGGCTTGTTGCCTCGTACGGGAAAATCTTGCCGTCTGCGATGGGTCAACAAACTCAAGCC<br>GGATCTCAAGAGAAGTGCCTTTTCCCCCGAGGAAGAGAAATTGGTAGTGGAGATGCAGGCGAAGCTTGGTAACAAGTGGGCAAAAATTGCTT<br>CATGTCTTCCTGGACGTACCGACAACGAAATTAAGAACTACTGGAACACTCGGATCAAGCGGAGGTTGCGCGCACTGCATAACTCCTCTGATG<br>CA
Chimera 7 Nter for trans-activation	ATGAAAACGATTCAAAACGGAGATGAAGTTCTACGGAAGGGTCCATGGATGCCCGAAGAAGACGAAATCCTGGTGGAGTATGTGCGACAATTT<br>GGTGAAGGAAACTGGAACTCGGTGCAGAAGCATTCGGGCTTGCCTCGTACGGGAAAATCTTGCCGTCTGCGATGGGTCAACAAACTCAAGC<br>CGGATCTCAAGAGAAGTGCCTTTTCCCCCGAGGAAGAGAAATTGGTAGTGGAGATGCAGGCGAAGCTTGGTAACAAGTGGGCAAAAATTGCT<br>TCATGTCTTCCTGGACGTACCGACAACGAAATTAAGAACTACTGGAACACTCGGATCAAGCGGAGGTTGCGCGCACTGCATAACTCCTCTGAT<br>GCA
At*DUO1* MYB for protein-Chip	GGGGACAAGTTTGTACAAAAAAGCAGGCTCCATGGAAGCCAAAAAAGAAGAGATCAAAAAAGGTCCGTGGAAAGCCGAAGAAGATGAAGTTC<br>TGATTAACCACGTTAAACGTTATGGTCCGCGTGATTGGAGCAGCATTCGTAGCAAAGGTCTGCTGCAGCGTACCGGTAAAAGCTGTCGTCTGC<br>GTTGGGTTAATAAACTGCGTCCGAATCTGAAAAACGGCTGTAAATTTTCAGCAGATGAAGAACGTACCGTGATTGAACTGCAGAGCGAATTTG<br>GTAATAAATGGGCACGTATTGCAACCTATCTGCCTGGTCGTACCGATAATGATGTTAAAAACTTTTGGAGCAGTCGTCAGAAACGTCTGGCACG<br>CATTTAAACCCAGCTTTCTTGTACAAAGTGGTCCCC
Mp*DUO1* MYB for protein-Chip	GGGGACAAGTTTGTACAAAAAAGCAGGCTCCATGAAAACCATTCAGAACGGTGATGAAGTTCTGCGTAAAGGTCCGTGGATGCCGGAAGAAG<br>ATGAAATTCTGGTTGAATATGTTCGTCAGTTTGGTGCACGTGATTGGAGCAGCATTCGTAGCAAAGGTCTGCTGCCTCGTACCGGTAAAAGCT<br>GTCGTCTGCGTTGGGTTAATAAACTGAAACCGGATCTGAAACGTAGCGGCTGTAAATTTAGCCCTGAAGAAGAAAAACTGGTCGTTGAAATGC<br>AGGCAAAACTGGGTAATAAATGGGCAAAAATTGCAAGCTGTCTGCCTGGTCGTACCGATAATGATGTTAAAAACTTTTGGAGCACCCGTCAGAA<br>ACGTATTCTGCGTGCATAAACCCAGCTTTCTTGTACAAAGTGGTCCCC
Chimera 4 MYB for protein-Chip	GGGGACAAGTTTGTACAAAAAAGCAGGCTCCATGAAAACCATTCAGAACGGTGATGAAGTTCTGCGTAAAGGTCCGTGGATGCCGGAAGAAG<br>ATGAAATTCTGGTTGAATATGTTCGTCAGTTTGGTGCACGTGATTGGAGCAGCATTCGTAGCAAAGGTCTGCTGCCTCGTACCGGTAAAAGCT<br>GTCGTCTGCGTTGGGTTAATAAACTGAAACCGGATCTGAAACGTAGCGCATTTAGCCCTGAAGAAGAAAAACTGGTTGTTGAAATGCAGGCCA<br>AACTGGGTAATAAATGGGCAAAAATTGCAAGCTGTCTGCCTGGTCGTACCGATAATGATGTTAAAAACTTTTGGAGCACCCGTCAGAAACGTAT<br>TCTGCGTGCATAAACCCAGCTTTCTTGTACAAAGTGGTCCCC
Chimera 5 MYB for protein-Chip	GGGGACAAGTTTGTACAAAAAAGCAGGCTCCATGAAAACCATTCAGAACGGTGATGAAGTTCTGCGTAAAGGTCCGTGGATGCCGGAAGAAG<br>ATGAAATTCTGGTTGAATATGTTCGTCAGTTTGGTGCACGTGATTGGAGCAGCATTCGTAGCAAAGGTCTGCTGCCTCGTACCGGTAAAAGCT<br>GTCGTCTGCGTTGGGTTAATAAACTGAAACCGGATCTGAAACGTAGCGGCTGTAAATTTAGCCCTGAAGAAGAAAAACTGGTCGTTGAAATGC<br>AGGCAAAACTGGGTAATAAATGGGCAAAAATTGCAAGCTGTCTGCCTGGTCGTACCGATAATGAAATCAAAAACTATTGGAACACCCGCATTAA<br>ACGTCGCCTGCGTGCATAAACCCAGCTTTCTTGTACAAAGTGGTCCCC
Chimera 6 MYB for protein-Chip	GGGGACAAGTTTGTACAAAAAAGCAGGCTCCATGAAAACCATTCAGAACGGTGATGAAGTTCTGCGTAAAGGTCCGTGGATGCCGGAAGAAG<br>ATGAAATTCTGGTTGAATATGTTCGTCAGTTTGGTGCACGTGATTGGAGCAGCATTCGTAGCAAAGGTCTGCTGCCTCGTACCGGTAAAAGCT<br>GTCGTCTGCGTTGGGTTAATAAACTGAAACCGGATCTGAAACGTAGCGCATTTAGCCCTGAAGAAGAAAAACTGGTTGTTGAAATGCAGGCCA<br>AACTGGGTAATAAATGGGCAAAAATTGCAAGCTGTCTGCCTGGTCGTACCGATAATGAAATCAAAAACTATTGGAACACCCGCATTAAACGTCG<br>CCTGCGTGCATAAACCCAGCTTTCTTGTACAAAGTGGTCCCC

Mp*MYB13* MYB for protein-Chip	GGGGACAAGTTTGTACAAAAAAGCAGGCTCCATGGAAACCATGGAACTGGGTAGCGCACCGGATTTTAGCGAAGATGTTGGTGCACTGAAAA<br>AAGGTCCGTGGACCAGCGCAGAAGATGCAATTCTGGTTGCATATGTTACCAAACATGGTGAAGGTAATTGGAACAGCGTTCAGAAACATTCAG<br>GTCTGTATCGTTGTGGTAAAAGCTGTCGTCTGCGTTGGGCAAATCATCTGCGTCCGAATCTGAAAAAAGGCGCATTTACACCGGAAGAGGAAC<br>GCATGATTATTGAACTGCATGCAAAACTGGGTAATAAATGGGCACGTATGGCAGCACAGCTGCCTGGTCGTACCGATAATGAAATCAAAAACTA<br>TTGGAACACCCGCATCAAACGTCGTATGCGTGCATAAACCCAGCTTTCTTGTACAAAGTGGTCCCC
Kfl*MYB* MYB for protein-Chip	GGGGACAAGTTTGTACAAAAAAGCAGGCTCCCCGAGCGAAAGCTGTAGCAGCGCAAGCAGCGAAGTTCAGCAGGCAGATGCCGGTGGTCT<br>GGTTAAAGGTGCATGGTCAACCGAAGAAGATGCACTGCTGCTGCAGTATGTTCAAGAACATGGTAGCAAAAATTGGGGCAATATTCAGCGTGT<br>TATCCGTGGTTTTAAACGTTGTGGTAAAAGCTGTCGTCTGCGTTGGGTTAATCATCTGCGTGGTGGTCTGAAAAAAACCGCACTGAGCCGTGA<br>TGAAGAACGTCGTGTTCTGGAACTGCATGCAACCCATGGTAATAAATGGGCCAAAATTGCAGCAGAACTGCCTGGTCGTACCGATAATAAAGTT<br>AAAAACTTTTGGAACGGTCGCCAGAAACGTATTATTCGTGCATAAACCCAGCTTTCTTGTACAAAGTGGTCCCC
Cpe*DUO1* MYB for protein-Chip	GGGGACAAGTTTGTACAAAAAAGCAGGCTCCAGCATGGGTGGCACCGCAACCGGTGGTGATAGCAGCGATCCGGAAGAACCGGGTCTGGC<br>AAAAGGCACCTGGCTGCCGGAAGAGGATCGTGTTCTGATGCAGTATGTTGGTAGCTATGGTCCGCGTAATTGGGGTGCACTGCGTGCACGTG<br>GTCTGCTGCGTCGTAGCGGTAAAAGCTGTCGTCTGCGTTGGGTTAATCAGCTGAAACCTGGTCTGCAGCGTTATGCAGGTAAAGGTTGTAAA<br>CGTTTTACCGAAGCCGAAGCAAATGTTGTTCTGAGCCTGCAGCGTGTTATGGGTAATAAATGGGCACAGATTAGCCGTCATCTGCCTGGTCGT<br>ACCGATAATGATGTTAAAAACTTTTGGAACCTGCACCTGAAACGTCAGGCACGTCTGTAAACCCAGCTTTCTTGTACAAAGTGGTCCCC
Cbr*DUO1* MYB for protein-Chip	GGGGACAAGTTTGTACAAAAAAGCAGGCTCCGGTAAAGGTGTTAGCGGTGGTGCACCGGCAGTTGGTCCGAAACGTCCGCTGAAACGTGGT<br>GTTTGGCTGCCGGAAGAAGATGAAATTCTGAAAGGTTATGTTGCAAACCAGGGTCCGAAAAATTGGAGCAGCATTGAAACCATGGGTCTGCT<br>GGCACGTAGCGGTAAAAGCTGTCGTCTGCGTTGGATGAATCATCTGCGTCCGGATCTGAATCGTCGTAGCACCAAATTTACCCCTGAAGAAGA<br>AGCAATCGTTGTTACCAAACAGAAACTGCATGGTAATAAATGGGCACAGATTGCAAAAAGCCTGAGCGGTCGTACCGATAATGATGTTAAAAAC<br>TTTTGGAATATGCGCATGAAAAAACTGGCCAAACTGGCGTAAGACCCAGCTTTCTTGTACAAAGTGGTCCCC

Supplementary Table 4. List of genes analyzed in this study.

Group	Species	Gene ID	Gene name	Name used in this study	Source
R2R3 MYB	*Marchantia polymoprha*	Mapoly0001s0061			phytozome.jgi.doe.gov
		Mapoly0006s0226			
		Mapoly0007s0265			
		Mapoly0007s0271			
		Mapoly0008s0029			
		Mapoly0019s0071	Mp*DUO1*	Mp*DUO1*	
		Mapoly0023s0101		Mp*R2R3-MYB07*/Mp*MYB14*	
		Mapoly0024s0094			
		Mapoly0032s0153			
		Mapoly0034s0034			
		Mapoly0056s0127			
		Mapoly0072s0075			
		Mapoly0073s0038			
		Mapoly0085s0092			
		Mapoly0096s0058			
		Mapoly0123s0012			
		Mapoly0318s0001			
		Mapoly0874s0001			
		Mapoly1089s0002		Mp*R2R3-MYB21*/Mp*MYB13*	
	Arabidopsis thaliana				www.arabidopsis.org, Ref. 5
	Klebsormidium flaccidum	kfl00011_0100			Ref. 6
		kfl00026_0540			
		kfl00040_0130			
		kfl00043_0140			
		kfl00048_0440			
		kfl00052_0040			
		kfl00064_0290			
		kfl00085_0320			
		kfl00097_0190			
		kfl00224_0130			
		kfl00241_0100		Kfl*MYB11*	
		kfl00265_0090		Kfl*MYB12*	
		kfl00279_0100			
		kfl00455_0010			
		kfl00455_0030			
		kfl00493_0040		Kfl*MYB16*	

		kfl00565_0070			
		kfl00698_0010			
		kfl00793_0050			
		kfl00861_0020			
		kfl00937_0030			
DUO1	*Solanum lycopersicum*	Solyc01g090530		Sly*DUO1A*	phytozome.jgi.doe.gov
		Solyc10g019260		Sly*DUO1B*	
	Vitis vinifera	GSVIVT01018234001		Vvi*DUO1*	
	Phaseolus vulgaris	Phvul.010G053200		Pvu*DUO1*	
	Medicago truncatula	Medtr8g006470		Mtr*DUO1*	
	Populus trichocarpa	Potri.014G054700		Ptr*DUO1*/Ptr*DUO1A*	
		Potri.002G140900		Ptr*DUO1B*	
	Aquilegia caerulea Goldsmith	Aquca_002_00029		Aca*DUO1*	
	Amborella trichopoda	LOC18442583	*MYB5*	Atr*DUO1*	www.ncbi.nlm.nih.gov
	Arabidopsis thaliana	AT3G60460	*DUO POLLEN1*	At*DUO1*	www.arabidopsis.org
	Brachypodium distachyon	Bradi5g17600		Bdi*DUO1*	phytozome.jgi.doe.gov
	Brachydodium stacei	Brast09G163900		Bst*DUO1*	
	Oryza sativa	LOC_Os04g46384		Osa*DUO1*	
	Zea mays	GRMZM2G105137		Zma*DUO1*	
	Picea abies	MA_130648g0010		Pab*DUO1*	congenie.org
	Selaginella moellendorffii	59725		Smo*DUO1*/Smo*DUO1A*	phytozome.jgi.doe.gov
	Physomitrella patens	Pp3c8_16720		Ppa*DUO1A*	
	Sphagnum fallax	Sphfalx0015s0253		Sfa*DUO1*	
	Marchantia polymorpha	Mapoly0019s0071		Mp*DUO1*	
	Spirogyra pratensis	comp6032_c0_seq2		Spr*DUO1*	Ref. 7
	Penium margaritaceum	comp156602_c0_seq1		Pma*DUO1*	Ref. 7
	Closterium peracerosum	LC172179		Cpe*DUO1*	in this study
	Mougeotia scalaris	comp14204_c0_seq4		Msc*DUO1*	Ref. 7
	Chara braunii	LC199499		Cbr*DUO1*	in this study
	Chara australis	LC221833		Cau*DUO1*	in this study
	Chara leptospora	LC221832		Cle*DUO1*	in this study
	Haplomitrium mniodes	submitted to DDBJ		Hmn*DUO1*	in this study
S18 MYB	*Amborella trichopoda*	LOC18445712		Atr*GAMYB1*	www.ncbi.nlm.nih.gov
		LOC18442852		Atr*GAMYB2*	
	Arabidopsis thaliana	AT5G06100	*ATMYB33*	At*MYB33*	www.arabidopsis.org
		AT3G11440	*ATMYB65*	At*MYB65*	
		AT4G26930	*ATMYB97*	At*MYB97*	
		AT2G32460	*ATMYB101*	At*MYB101*	
		AT5G55020	*ATMYB120*	At*MYB120*	

	Brachypodium distachyon	Bradi1g32720		Bdi*GAMYB*	phytozome.jgi.doe.gov
	Marchantia polymorpha	Mapoly1089s0002		Mp*R2R3-MYB21*	
		Mapoly0023s0101		Mp*R2R3-MYB07*	
	Oryza sativa	LOC_Os06g46560		Osa*GAMYB*	
	Physcomitrella patens	PP1S66_200V6		Ppa*GAMYB1*	plants.ensembl.org
		PP1S238_71V6		Ppa*GAMYB2*	
	Picea abies	MA_20462g0010		Pab*GAMYB1*	congenie.org
		MA_96853p0010		Pab*GAMYB2*	
		MA_10208000g0010		Pab*GAMYB3*	
	Populus trichocarpa	Potri.001G036000		Ptr*GAMYB1*	phytozome.jgi.doe.gov
		Potri.002G228700		Ptr*GAMYB2*	
		Potri.003G189700		Ptr*GAMYB3*	
		Potri.009G018700		Ptr*GAMYB4*	
	Selaginella moellendorffii	SELMODRAFT_81112		Smo*GAMYB1*	plants.ensembl.org
		SELMODRAFT_84369		Smo*GAMYB2*	
	Sphagnum fallax	Sphfalx0019s0143		Sfa*GAMYB1*	phytozome.jgi.doe.gov
		Sphfalx0221s0015		Sfa*GAMYB2*	
		Sphfalx0126s0030		Sfa*GAMYB3*	
	Vitis vinifera	GSVIVG01030434001		Vvi*GAMYB1*	
		GSVIVT01012447001		Vvi*GAMYB2*	
DAZ	*Arabidopsis thaliana*	AT2G17180	*DAZ1*	At*DAZ1*	www.arabidopsis.org
		AT4G35280	*DAZ2*	At*DAZ2*	
	Salix purpurea	SapurV1A.0125s0370			phytozome.jgi.doe.gov
		SapurV1A.0374s0220			
		SapurV1A.0475s0070			
		SapurV1A.0530s0140			
		SapurV1A.3538s0020			
	Eucalyptus grandis	Eucgr.I01518			
	Medicago truncatula	Medtr5g062300			
		Medtr5g099040			
	Solanum lycopersicum	Solyc06g060480.1			
	Aquilegia caerulea Goldsmith	Aquca_015_00379			
	Zea mays	GRMZM2G132057			
	Oryza sativa	Os02g19200			
	Amborella trichopoda	scaffold00001.9			
	Selaginella moellendorffii	410896			
		424361			
	Physcomitrella patens	Pp3c22_2200			
		Pp3c19_21390			

		Pp3c21_440			
		Pp3c18_22010			
	Sphagnum fallax	Sphfalx0048s0033			
		Sphfalx0022s0120			
		Sphfalx0020s0104			
		Sphfalx0179s0034			
		Sphfalx0019s0188			
	Marchantia polymorpha	Mapoly0011s0122	Mp*DAZ1*	Mp*DAZ1*	in this study
GCS1/HAP2	*Arabidopsis thaliana*	AT4G11720	*AtGCS1/HAP2*	At*GCS1/HAP2*	www.arabidopsis.org
	Salix purpurea	SapurV1A.0507s0190			phytozome.jgi.doe.gov
	Eucalyptus grandis	Eucgr.D01915			
	Medicago truncatula	Medtr5g010740			
	Solanum lycopersicum	Solyc08g080450.1			
	Aquilegia caerulea Goldsmith	Aquca_013_00398			
	Zea mays	GRMZM2G412911			
	Oryza sativa	LOC_Os05g18730			
		LOC_Os09g35720			
	Amborella trichopoda	scaffold00069.68			
		scaffold00069.69			
	Selaginella moellendorffii	XP_2971451.1			
	Physcomitrella patens	Pp3c25_15230			
	Marchantia polymorpha	Mapoly0066s0032	Mp*GCS1*	Mp*GCS1*	in this study
GEX2	*Arabidopsis thaliana*	AT5G49140	*AtGEX2*	At*GEX2*	www.arabidopsis.org
	Salix purpurea	SapurV1A.1573s0050			phytozome.jgi.doe.gov
	Eucalyptus grandis	Eucgr.H03956			
	Medicago truncatula	Medtr2g436170			
		Medtr2g435850			
		Medtr5g020160			
	Solanum lycopersicum	Solyc01g066510.1			
	Aquilegia caerulea Goldsmith	Aquca_003_00691			
	Zea mays	GRMZM2G036832			
	Oryza sativa	LOC_Os09g25650			
	Amborella trichopoda	scaffold00019.109			
	Selaginella moellendorffii	416480			
		407438			
		412212			
	Marchantia polymorpha	Mapoly0125s0024	Mp*GEX2*	Mp*GEX2*	in this study

Supplementary References

1. Chen C, Barfuss MH, Proschold T, Schagerl M. Hidden genetic diversity in the green alga Spirogyra (Zygnematophyceae, Streptophyta). *BMC Evol Biol* **12**, 77 (2012).

2. Lemieux C, Otis C, Turmel M. Comparative Chloroplast Genome Analyses of Streptophyte Green Algae Uncover Major Structural Alterations in the Klebsormidiophyceae, Coleochaetophyceae and Zygnematophyceae. *Front Plant Sci* **7**, 697 (2016).

3. She R, Jarosz DF. Mapping Causal Variants with Single-Nucleotide Resolution Reveals Biochemical Drivers of Phenotypic Change. *Cell* **172**, 478-490 e415 (2018).

4. Higo A, *et al.* Transcriptional Framework of Male Gametogenesis in the Liverwort Marchantia polymorpha L. *Plant Cell Physiol* **57**, 325-338 (2016).

5. Du H, *et al.* The Evolutionary History of R2R3-MYB Proteins Across 50 Eukaryotes: New Insights Into Subfamily Classification and Expansion. *Sci Rep* **5**, 11037 (2015).

6. Hori K, *et al.* Klebsormidium flaccidum genome reveals primary factors for plant terrestrial adaptation. *Nat Commun* **5**, 3978 (2014).

7. Cooper E, Delwiche C. Green algal transcriptomics for phylogenetics and comparative genomics. *figshare*, https://dx.doi.org/10.6084/m6089.figshare.1604778 (2016).
