# Supplementary material for: Transcription factor DUO1 generated by neo-functionalization is associated with evolution of sperm differentiation in plants
Source: Nat Commun. 2018 Dec 11;9:5283. doi: 10.1038/s41467-018-07728-3 (PMC6290024; doi:10.1038/s41467-018-07728-3)
Supplement: Supplementary file 2 — Description of Additional Supplementary Files [file 41467_2018_7728_MOESM2_ESM.pdf]

Description of Additional Supplementary Files

File Name: Supplementary Movie 1
Description: Movement of Marchantia Wild-Type Sperm Discharged into Water. The movie of discharged sperm of Mpmid-1ko plant was taken at the rate of 20 frames per second (fps) for 6 sec by a microscope camera DP26 (OLYMPUS).

File Name: Supplementary Movie 2
Description: Movement of Marchantia Mpmid-1ko Sperm Discharged into Water. The movie of discharged sperm of WT plant was taken at the rate of 4 frames per second (fps) for 30 sec by a microscope camera DP26 (OLYMPUS).
